# Blimp1 Activation by AP-1 in Human Lung Cancer Cells Promotes a Migratory Phenotype and Is Inhibited by the Lysyl Oxidase Propeptide

**DOI:** 10.1371/journal.pone.0033287

**Published:** 2012-03-15

**Authors:** Ziyang Yu, Seiichi Sato, Philip C. Trackman, Kathrin H. Kirsch, Gail E. Sonenshein

**Affiliations:** 1 Department of Biochemistry, Tufts University School of Medicine, Boston, Massachusetts, United States of America; 2 Division of Oral Biology, Boston University Henry M. Goldman School of Dental Medicine, Boston, Massachusetts, United States of America; 3 Department of Biochemistry, Boston University School of Medicine, Boston, Massachusetts, United States of America; Cincinnati Children's Hospital Medical Center, United States of America

## Abstract

B lymphocyte-induced maturation protein 1 (Blimp1) is a master regulator of B cell differentiation, and controls migration of primordial germ cells. Recently we observed aberrant Blimp1 expression in breast cancer cells resulting from an NF-κB RelB to Ras signaling pathway. In order to address the question of whether the unexpected expression of Blimp1 is seen in other epithelial-derived tumors, we selected lung cancers as they are frequently driven by Ras signaling. Blimp1 was detected in all five lung cancer cell lines examined and shown to promote lung cancer cell migration and invasion. Interrogation of microarray datasets demonstrated elevated *BLIMP1* RNA expression in lung adenocarcinoma, pancreatic ductal carcinomas, head and neck tumors as well as in glioblastomas. Involvement of *Ras* and its downstream kinase c-Raf was confirmed using mutant and siRNA strategies. We next addressed the issue of mechanism of Blimp1 activation in lung cancer. Using knockdown and ectopic expression, the role of the Activator Protein (AP)-1 family of transcription factors was demonstrated. Further, chromatin immunoprecipitation assays confirmed binding to identified AP-1 elements in the *BLIMP1* promoter of ectopically expressed c-Jun and of endogenous AP-1 subunits following serum stimulation. The propeptide domain of lysyl oxidase (LOX-PP) was identified as a tumor suppressor, with ability to reduce Ras signaling in lung cancer cells. LOX-PP reduced expression of Blimp1 by binding to c-Raf and inhibiting activation of AP-1, thereby attenuating the migratory phenotype of lung cancer cells. Thus, Blimp1 is a mediator of Ras/Raf/AP-1 signaling that promotes cell migration, and is repressed by LOX-PP in lung cancer.

## Introduction

B lymphocyte-induced maturation protein 1 (Blimp1) or Positive-Regulatory Domain I Binding Factor 1 (PRDI-BF1) is a zinc finger protein encoded by the *PRDI-BF1 and RIZ domain 1* (*PRDM1*) or *BLIMP1* gene [1], [2], which was initially isolated as a transcriptional repressor of the *IFNβ* promoter [3]. Several mechanisms of Blimp1-mediated repression of gene transcription have been elucidated: recruitment of histone methyltransferases (HMTs) [4], histone deacetylases (HDACs) [5] or corepressors [2] or by competition with transcriptional activators [6]. Blimp1 was identified as a master regulator of B cell terminal differentiation [7], which promotes differentiation of B lymphocytes to plasma cells [8]. Several factors have been implicated in the activation of transcription of the *Blimp1* gene during the differentiation of B cells, including NF-κB, AP-1, IRF4, STAT3 and STAT5, although, their precise mechanisms of action are not fully understood [9]. Blimp1 was subsequently shown to regulate T cell proliferation and homeostasis [10]. During development, Blimp1 controls primordial germ cell (PGC) specification and migration as Blimp1-deficient mouse embryos generate PGC-like cells which fail to show characteristic PGC migration [11], [12]. Somewhat unexpectedly, Blimp1 was detected in non-hematopoietic cancer cells. Our laboratory observed Blimp1 expression in breast cancer cells, and showed it repressed transcription of the *ESR1* gene encoding estrogen receptor alpha (ERα), thereby promoting a more migratory phenotype [13]. Transcriptional induction of Bcl-2 levels by the NF-κB RelB subunit recruited Ras to the mitochondria [14]. The resultant Ras signaling led to an aberrant induction of Blimp1 in the breast cancer cells [13]. The exact transcription factor(s) downstream of Ras that mediated the activation of Blimp1 in these cancer cells remained to be identified. However, the involvement of Ras signaling in Blimp1 activation leads us to hypothesize that expression of Blimp1 may be more widespread in cancer than previously realized. Colorectal tumor cells were also found to express Blimp1, which repressed the *TP53* gene and thus maintained cell growth [15].

Lung cancer is the leading cause of cancer-related death in Western countries. Approximately two-thirds of patients are diagnosed at an advanced stage, and of the remaining patients who undergo surgery, 30–50% develop recurrence with metastatic disease [16], [17]. The *RAS* oncogene is mutated in up to ∼30% of lung cancers, with the majority of mutations found in the *KRAS* gene [16], [17]. Oncogenic K-Ras predisposes transgenic mice to lung tumorigenesis [18]. Ras signals via multiple pathways, including mitogen activated protein kinase (MAPK). As nuclear acceptors for MAPK signaling cascades, the activator protein (AP)-1 family of transcription factors has been implicated in the highly migratory phenotype of lung cancer cells [19], [20], [21].

The *lysyl oxidase* (*LOX*) gene was isolated as the *ras recision gene* (*rrg*) due to its ability to revert Ras-mediated transformation of NIH 3T3 fibroblasts [22]. Our group showed ectopic Pro-LOX expression reduced extracellular signal-regulated kinase (ERK) and phosphatidylinositol 3-kinase (PI3K)/Akt signaling and activation of NF-κB in Ras-transformed NIH 3T3 cells [23]. Loss of *LOX* gene expression was seen in many cancerous tissues and derived cell lines including those from lung [24], [25], [26], colon [27], prostate [28], gastric [29] and head and neck squamous cancers [30]. Ectopic *LOX* gene expression reduced colony formation of cultured gastric cancer cells and tumor formation in a xenograft model [29]. Lysyl oxidase is synthesized and secreted as a pro-enzyme (Pro-LOX), and processed to a functional enzyme (LOX) and amino terminal propeptide (LOX-PP) [31]. The *rrg* activity of Pro-LOX was unexpectedly mapped to the LOX-PP domain, as judged by inhibition of the transformed phenotype of NIH 3T3-Ras cells [32]. Subsequently, LOX-PP was shown to reduce the migratory phenotype of mouse breast cancer cells driven by Her-2/Neu, which signals via Ras and their ability to form tumors in a nude mouse xenograft model [33], [34]. In H1299 lung cancer cells, which contain a mutant *NRAS* gene, LOX-PP reduced the activation of ERK and Akt, and ability for anchorage-independent growth and invasive colony formation in Matrigel [25]. LOX-PP also attenuated fibronectin-mediated activation of focal adhesion kinase in breast cancer cells [34], [35], and fibroblast growth factor (FGF)-2-induced proliferation of prostate cancer cells [36]. Here we asked whether Blimp1 is expressed in lung cancer cells given the important role of Ras signaling in these cancer cells. Blimp1 was detected in all lung cancer lines examined and promoted their migration and invasion. Furthermore, *BLIMP1* RNA was detected in other primary tumors driven by Ras signaling. In lung cancer cells, Blimp 1 expression was induced by a Ras/c-Raf/AP-1 pathway, which could be inhibited by LOX-PP via interaction with c-Raf. Thus, these studies identify Blimp1 as a critical mediator of lung cancer cell migratory phenotype by the transforming Ras/c-Raf/AP-1 cascade.

## Materials and Methods

### Cells and culture conditions

The non-small cell lung cancer (NSCLC) A549 and H1299 cell lines were kindly provided by Zhi-Xiong Jim Xiao (Boston University School of Medicine, Boston MA). The Calu-1, H23 and H441 cell lines were generously provided by Hasmeena Kathuria and Maria Ramirez (Boston University School of Medicine). A549, Calu-1, H23 and H441 cells express mutant K-Ras [37], [38] and H1299 express mutant N-Ras [39]. Bosc23 cells were obtained from the American Type Culture Collection (ATCC). All cell lines were maintained in Dulbecco's Minimal Essential Medium except H441 which was maintained in RPMI-1640. The culture media were supplemented with 10% fetal bovine serum (FBS), as recommended by the ATCC. H1299 clones expressing mouse LOX-PP in a doxycycline (dox) inducible vector were established and total RNA isolated as described previously [25]. Inducible stable A549 cells expressing V5-tagged human or mouse LOX-PP were established as previously described [33], [34]. Briefly, pCL-Ampho retrovirus packaging vector (Imgenex, San Diego, CA) was co-transfected into BOSC 23 cells using FuGENE 6 (Roche Diagnostics Co., Indianapolis, IN) with either empty effector vector pC4_bsr_R(TO) (EV) or vector bearing the DNA fragments of human or mouse LOX-PP with C-terminal V5 tag and the regulator vector pCX_neo_TR2 (both kindly provided by Tsuyoshi Akagi, KAN, Kobe, Japan). After 48 h, supernatants containing viral particles were harvested and passed through a 0.45 µm filter (Corning Inc., Corning, NY). A549 lung cancer cells were dually infected for 48 h with supernatant from BOSC 23 cells containing viruses that carry the regulator and effector vectors supplemented with 6 µg/ml polybrene (Sigma, St. Louis, MO). Infected cells were selected with 10 µg/ml blasticidin (Invitrogen, Carlsbad, CA) and 1.4 mg/ml geneticin (Sigma) to generate separate pools of stable A549-EV, A549-human LOX-PP and A549-mouse LOX-PP cells.

### Plasmids and transfection analysis

The pcDNA3/Blimp [2] and the 7-kB *Blimp1*-pGL3 luciferase reporter (*Blimp1*-luc) [40] vectors were kindly provided by Tom Maniatis (Columbia University, NY) and Kathryn Calame (Columbia University), respectively. The c-Jun, c-Fos, Fra-1 and Fra-2 AP-1 constructs in pCI expression vector were as previously reported [41]. For transient transfection of expression vectors, cultures in 12-well plates were incubated for 48 h in the presence of 1 µg DNA and 3 µl Fugene 6 or 2.5 µl Lipofectamine 2000 (Invitrogen). Co-transfection of the MSV-β-gal vector, expressing β-galactosidase (β-gal) was used to normalize for transfection efficiency. All transient transfection reporter assays were performed, in triplicate, two times as described previously [42], and the standard error of the mean (SEM) calculated. *BLIMP1* siRNA, and *JUN*, *FRA-1* and *FRA-2* siRNA duplex sequences were as previously described [15], [43]. The siRNA targeting human *KRAS* gene (sc-35731) was from Santa Cruz Biotechnology (Santa Cruz, CA). The RNA duplexes used for targeting *c-RAF* were as described by Chadee and Kyriakis [44] and purchased from QIAGEN (Valencia, CA). For transient transfection of single siRNAs, cultures in 6-well plates were incubated for 24 h in the presence of siRNA duplex (10 nM final) and Lipofectamine RNAiMax (Invitrogen), according to the manufacturer's protocol. In the case of co-transfection of two AP-1 siRNAs, the final concentration of each siRNA was 10 nM, making the total siRNA concentration 20 nM. Where mentioned, the culture was supplemented with a negative control siRNA (Qiagen) at a final concentration of either 10 or 20 nM, as appropriate. The Ras S186 expression vector was kindly provided by Mark Philips (NYU School of Medicine, New York, NY). For construction of N-terminally glutathione *S*-transferase (GST) tagged LOX-PP and its deletion mutants, the cDNA encoding full length LOX-PP (WT, amino acid 1–162) and deletion of aa residues 26–100 (ΔM3) were amplified from full-length Pro-LOX cDNA [33] and inserted into the BamHI/ClaI site of pEBG-GST mammalian expression vector, a generous gift of Dr. Bruce Mayer (University of Connecticut Health Center, Farmington, CT). For construction of C-terminally GST-tagged LOX-PP, the cDNAs encoding GST and LOX-PP were amplified and inserted into pcDNA3.1 (+). pBabe-puro-MEK1 S217E/S221E constitutively active (CA-MEK) mutant was kindly supplied by Dr. Geoffrey M. Cooper (Boston University, Boston, MA). The cDNA encoding MEK1 S217E/S221E was inserted into pcDNA3.1(+).

### Immunoblot analysis

Nuclear extracts (NE) and whole cell extracts (WCE) were prepared and subjected to immunoblotting, as described previously [33]. For the detection of secreted recombinant LOX-PP-V5, culture medium (40 µl from 2 ml of culture medium) was immunoblotted using an anti-V5 antibody (R960-25, Invitrogen). The antibodies against Blimp1 (no. 9115s), c-Jun (no. 9165), phospho-c-Jun (no. 9261s), MEK1/2 (L38C12; no. 4694) phospho-ERK1/2 (phospho-Thr202/Tyr204; no. 9101s) and ERK1/2 (9102) were obtained from Cell Signaling (Danvers, MA). Antibodies against GST (B-14), K-Ras (F234), B-Raf (F-7), Fra-1 (N-17), Fra-2 (Q-20) and c-Fos (H-125) were from Santa Cruz Biotechnology. Antibodies against β-actin (AC-15) and α-tubulin (DM1A) were from Sigma. Hsp70/Hsc70 (SPA-820) and Hsp90 (SPA-830) antibodies were purchased from Stressgen (Victoria, BC, Canada). Antibody against c-Raf (clone 53) and Ras (clone 18/Ras) were from BD Transduction (Franklin Lakes, NJ). Rabbit polyclonal antibodies against LOX-PP were prepared as described previously [45]. The results from a minimum of two independent experiments were subjected to densitometry and normalized to a β-actin loading control and the mean values relative to control empty vector (EV) cells (set to 1.0) given.

### Migration and invasion assays

Suspensions of 1×10^5^ cells were layered, in triplicate, in the upper compartments of Costar Transwells (Corning, Lowell, MA) on an 8-mm diameter polycarbonate filter (8 µm pore size), and incubated at 37°C for 16 h. Migration of the cells to the lower side of the filter was evaluated with the phosphatase enzymatic assay using p-nitrophenyl phosphate and OD_410 nm_ determination, as described previously [13] or by staining with crystal violet and OD_570 nm_ determination (63). The average migration from three independent experiments ± SD is presented relative to the control EV, which was set at 1.0. *P* values were calculated using a Student's *t*-test. For invasion assays, filters were precoated with 10 µg of Matrigel (BD Biosciences, San Jose, CA). Migration of the cells to the lower side of the filter was evaluated by staining with crystal violet and OD_570 nm_ determination. The mean ± SD are presented. Invasion assays were performed three times, in triplicate.

### Reverse Transcriptase (RT)-PCR analysis

RNA was isolated using RNeasy Mini Kit (Invitrogen), and samples with A_260_/A_280_ ratios between 1.8 and 2.0 were treated with RQ1 RNase-free DNase (Promega). Superscript III RT was used for reverse transcription with 1 µg RNA in the presence of 100 ng of random primers (Invitrogen). For Realtime quantitative PCR (Q-PCR), the *BLIMP1* primers were as described previously [46]. The *GAPDH* primers were: Forward 5′-TTGCCATCAATGACCCCTTCA-3′; Reverse 5′-CGCCCCACTTGATTTTGGA-3′. Q-PCR was performed in triplicate in a Roche LightCycler 480 system.

### Chromatin Immunoprecipitation (ChIP) assay

ChIP assays were performed using an EZ-ChIP kit (Millipore Corporation, Billerica, MA), according to the manufacturer's instructions. For analysis of ectopically expressed AP-1, 24 h after H441 cells were transfected with a c-Jun expression vector, formaldehyde (1% final) was added to the cell culture medium. Whole cell lysates were made and subjected to sonication in a Misonix 3000 Sonicator (Misnonix, Farmingdale, NY) for 15 cycles of 10 sec each to yield genomic DNA fragments of ∼200 to 1000 bp. After preclearing with ChIP grade Protein G agarose, 100 µl of sheared DNA-protein complexes were immunoprecipitated with antibodies against c-Jun (sc-1694) or normal rabbit IgG (sc-2027) (Santa Cruz Biotechnology). Crosslinking was reversed and purified genomic DNA fragments were subjected to PCR. The crosslinking was reversed by overnight incubation at 65°C and genomic DNA fragments purified with a Qiaquick PCR purification kit (QIAGEN, no. 28104). Two binding elements for AP-1, which are also known as TPA responsive elements or TREs, were previously identified at −1813 and −1647 bp relative to the *BLIMP1* transcription start site [47] and verified using TransFac (genomatix.de) analysis. The region across the two TREs was amplified by PCR. The primers for the −1813 bp TRE: Forward 5′-GCCTTCTTCCCACCTCAAATATCA-3′, Reverse 5′-TGGCCTGCTGTTCAAACAGTCT-CA-3′; and for the −1647 bp TRE: Forward 5′-GTTGCATGATGGTGTATGTGGCCT-3′, Reverse 5′-ATCCAGCCTGCTCAAGAGGGTTTA -3′. As a positive control for AP-1 binding, a fragment of the human *JUN* promoter containing two closely located TRE sites (−120 and −1 bp) was similarly subjected to ChIP analysis, using the previously described primers [48]. As a negative control, primers were designed for an upstream region of the *BLIMP1* promoter (−5508 to −5366 bp) containing no TRE sites: Forward 5′- TCCTTCCCTGTGTTTGGTCCCATT-3′, Reverse 5′-ATTGTTTCCTTCAAGCAGGCACCC-3′. For binding of endogenous AP-1 subunits, A549 cells were incubated in serum-free medium for 48 h and FBS (10% final concentration) added back. After 30 min, WCE were prepared and subjected to ChIP assay, as above, using antibodies against normal rabbit IgG, c-Jun, Fra-1 (sc-183), or Fra-2 (sc-604) (from Santa Cruz Biotechnology).

### Immunoprecipitation and GST pull down assay

H1299 or A549 cells were lysed with Buffer A [25 mM HEPES-KOH (pH 7.2), 150 mM KCl, 2 mM EDTA, 1 mM phenylmethylsulfonyl fluoride, 1 mM dithiothreitol, 0.5 µg/ml leupeptin, 2 µM pepstatin A, 1 µg/ml aprotinin, and 1% Triton X-100]. The lysates were centrifuged in a microcentrifuge for 10 min at 13,000 rpm at 4°C to remove insoluble material. For immunoprecipitation, 2 µg of either rabbit anti-LOX-PP [45] or rabbit control IgG was added to 500 µg cell lysate, followed by overnight incubation at 4°C. Protein G-Sepharose beads (Invitrogen) were then added to the mixture, followed by incubation at 4°C for 2 h with gentle shaking. The beads were washed four times with Buffer A. For GST-pull down assay, the lysates were incubated with 20 µl Glutathione-Sepharose 4B (GE Healthcare) for 2 h at 4°C. The resin was washed four times with Buffer A. The immune-complexes or GST pull down-complexes were eluted from the Sepharose beads with SDS-PAGE sample buffer, and the precipitated proteins analyzed by immunoblot analysis.

## Results

### Lung cancer cells express Blimp1

**Figure 1:**
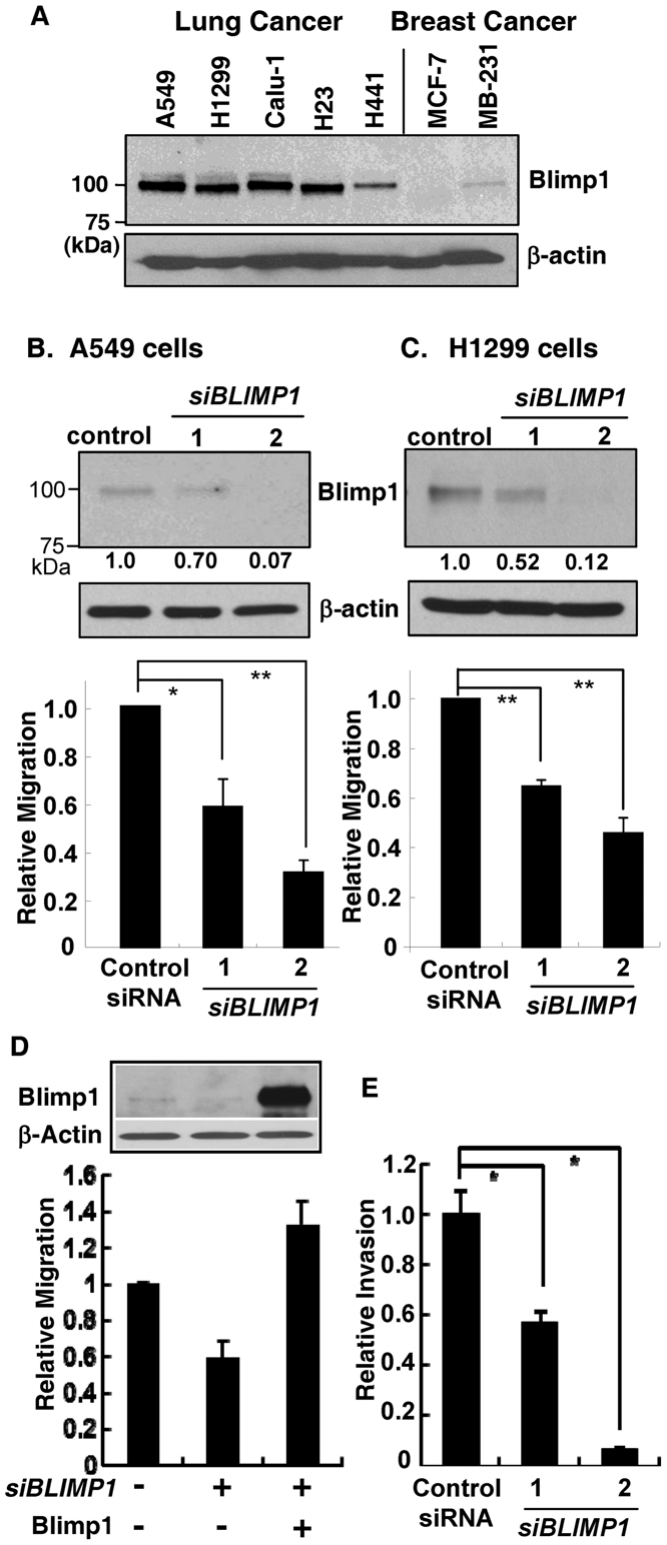
Blimp1 is expressed in lung cancer cells and its knockdown reduces migration. (A) Samples of nuclear extracts (20 µg) of A549, H1299, Calu-1, H23 and H441 human lung cancer cells and MCF-7 and MDA-MB-231 (MB-231) breast cancer cells were subjected to immunoblotting for Blimp1 and β-actin, as a control for equal loading. Positions of molecular weight markers are given in the left lane. A representative of two independent experiments with similar results is shown. (B) A549 and (C) H1299 cells were transiently transfected with 10 nM each of *siBLIMP1-1*, *siBLIMP1-2* or a scrambled negative control siRNA. Upper panels: Forty-eight h after transfection, WCE (30 µg) were subjected to immunoblotting for Blimp1 and β-actin. The bands were quantified using NIH Image J software and Blimp1 expression normalized to β-actin expression. Normalized Blimp1 expression was determined in two independent experiments and the average values are given below the blots. Lower panels: Alternatively, after 24 h, cultures were trypsinized and 1×10^5^ cells subjected to a migration assay for 16 h, in triplicate. The average migration from three independent experiments ± SD is presented relative to the negative control siRNA (set at 1.0). *P* values were calculated using Student's *t*-test. *, *P*<0.005; **, *P*<0.0005. (D) A549 cells were incubated in the presence of 0.5 nM *siBlimp1-2* or scrambled negative control siRNA for 16 h. Cells were then transfected with Blimp1 expression vector (2 µg per well in 6-well plate) and incubated for 32 h. (Inset) Whole cell lysates (20 µg) were subjected to western blot analysis using antibodies against Blimp1 or β-actin. Cultures were trypsinized and 1×10^5^ cells subjected to a migration assay for 16 h, in triplicate. The average migration from two independent experiments ± SE is presented relative to the negative control siRNA and EV (set at 1.0). Data shown is a representative of two independent experiments with similar results. (E) A549 cells were transiently transfected with 10 nM each of *siBLIMP1-1*, *siBLIMP1-2* or a scrambled negative control siRNA. After 48 h, cultures were trypsinized and 1×10^5^ cells subjected to an invasion assay for 16 h, in triplicate. The average data from three independent experiments ± SD is presented relative to the negative control siRNA (set at 1.0). *P* values were calculated using Student's *t*-test. *, P<0.01.

Five lung cancer cell lines, driven by mutant K-Ras or N-Ras, were selected to test for Blimp1 expression: A549, H1299, Calu-1, H23 and H441. Nuclear extracts were subjected to immunoblot analysis (Fig. 1A). As a reference, we included nuclear extracts from ERα positive MCF-7 and ERα negative MDA-MB-231 breast cancer cells, which displayed relatively lower and higher Blimp1 levels, respectively [13]. All five lung cancer cell lines expressed 100 kDa Blimp1 protein recognized by an antibody against the N-terminus of the human Blimp1 protein. As seen previously, the ERα negative MDA-MB-231 breast cancer cells expressed higher levels of Blimp1 than the ERα positive MCF-7 cells [13]. All of the lung cancer cells expressed substantially higher amounts of Blimp1 than the MDA-MB-231 line. Thus, Blimp1 is expressed in lung cancer cells.

### Blimp1 promotes migration of lung cancer cells

The absence of Blimp1 in mouse embryos led to development of primordial germ-like cells that were unable to migrate [11], [12]. To test whether Blimp1 expression is involved in control of lung cancer cell migration, a knockdown strategy was used. A549 and H1299 cells, which displayed relatively high levels of Blimp1 (Fig. 1A), were incubated with either *siBLIMP1-1* or *siBLIMP1-2*, two independent siRNA species, or with a scrambled negative control siRNA. After 48 h, samples of WCE were subjected to immunoblot analysis. Both *BLIMP1* siRNAs resulted in effective knockdown of Blimp1 protein expression compared to the control siRNA. A more robust knockdown was seen with *siBLIMP1-2* in both cell lines, i.e., 93% decrease in A549 and 88% in H1299 compared to 30% in A549 and 48% in H1299 with *siBLIMP1-1* (upper panels, Figs. 1B and 1C). The effects of a 24 h incubation with these siRNAs on migration of A549 and H1299 cells were tested in Boyden chambers (1×10^5^ per well) using FBS as the chemo-attractant. Cell migration was measured 16 h later. Knockdown of *BLIMP1* expression led to decreased migration of A549 (Fig. 1B) and H1299 (Fig. 1C) lung cancer cells. In three independent experiments, performed in triplicate, *siBLIMP1-2* led to a more profound reduction in migration of A549 (average decreases of 42% with *siBLIMP1-1* vs 71% with *siBLIMP1-2*) and H1299 cells (average decreases of 35% with *siBLIMP1-1* vs 54% with *siBLIMP1-2*). No significant effects of the treatments on cell proliferation were observed (data not shown). These results are consistent with the reduction of Blimp-1 levels. To confirm that the reduction of cell migration was specifically due to the knockdown of Blimp1 expression, we performed a rescue experiment using *siBLIMP1-2* and Blimp1 ectopic expression in A549 lung cancer cells. Briefly, A549 cells were incubated with either *siBLIMP1-2* or negative control siRNA for 16 h followed by transient transfection of a vector expressing Blimp1 or EV DNA. After 32 h, cells were subjected to a migration assay or samples of WCE were subjected to immunoblot analysis. *BLIMP1* siRNA-2 resulted in effective knockdown of Blimp1 protein expression compared to the control siRNA and this effect was overcome by the Blimp1 expression vector (Fig. 1D, inset). As seen above, knockdown of *BLIMP1* expression led to a 42% decrease in cell migration compared to cells transfected with negative control siRNA and EV, and this was overridden by ectopic Blimp1 expression (Fig. 1D). A 33% increase in migration of A549 cells transfected with Blimp1 cDNA and *siBlimp1-2* was observed compared to the control siRNA and EV transfected cells. In addition, no significant effects on cell proliferation were noted over the time course (data not shown). Next, we tested the effects of Blimp1 knockdown on invasion. A decrease in invasion by A549 lung cancer cells was noted with *BLIMP1* siRNA-1, which was even more profound with *BLIMP1* siRNA-2 compared to the negative control siRNA, consistent with the migration data (Fig. 1E). Thus, reduced levels of Blimp1 lead to decreased ability of A549 and H1299 lung cancer cells to migrate and invade.

**Figure 2:**
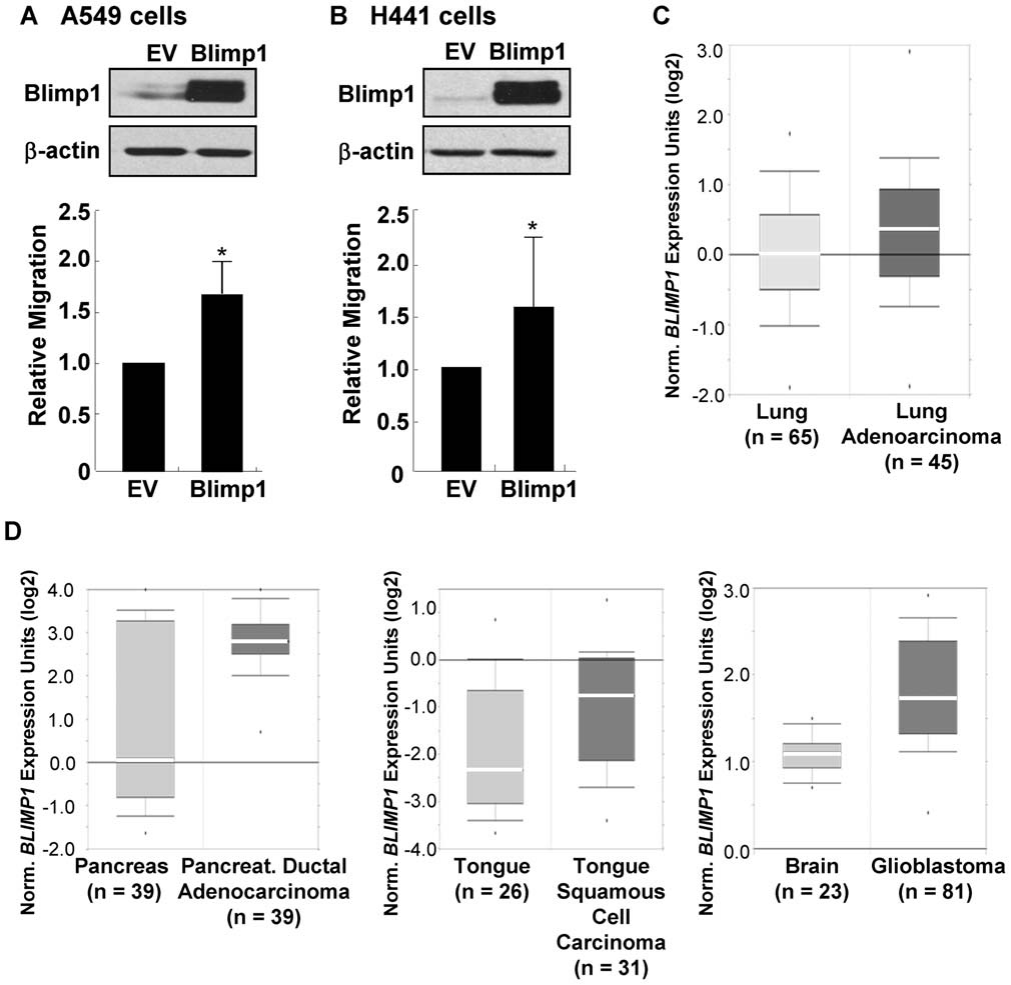
Blimp1 promotes lung cancer cell migration and is aberrantly expressed in multiple cancers. (A) A549 cells or (B) H441 cells were transiently transfected with 1 µg of Blimp1 cDNA or EV DNA using Lipofectamine 2000. Upper panels: WCE were isolated after 48 h and subjected to immunoblot analysis for Blimp1 and β-actin. Lower panels: Alternatively, 24 h after transfection, cells were subjected to a migration assay as in Fig. 1. The average migration from three independent experiments ± SD is presented relative to the EV (set at 1.0). *P* values were calculated using a Student's *t*-test. *, *P*<0.005. C) Box plot from the Hou lung cancer microarray dataset was accessed using Oncomine Database. Student's *t*-test for the two groups shows a *P* value of 0.024. D) Box plots from the Badea pancreatic cancer, Estilo head-neck cancer and Sun brain tumor microarray datasets were accessed using Oncomine Database. Student's *t*-tests comparing the groups in these studies show *P* values of 8.67e^−7^, 0.001 and 3.28e^−15^, respectively.

We next performed the converse experiment and ectopically expressed Blimp1 in A549 and H441 cells, which express higher and moderate levels of Blimp1, respectively. Cultures were transiently transfected with a Blimp1 expression cDNA or parental empty vector (EV) for 24 h and subjected to migration assays, as above. In three independent experiments, performed in triplicate, Blimp1 overexpression increased migration of A549 and H441 cells by an average of 64% (Fig. 2A) and 58% (Fig. 2B), respectively. Western blotting of extracts prepared from similarly transfected cultures confirmed ectopic expression of Blimp1 (upper panels, Figs. 2A and 2B). Thus, Blimp1 promotes a more migratory phenotype of lung cancer cells.

### Multiple primary tumors display overexpression of *BLIMP1* RNA

We next asked whether *BLIMP1* RNA is detected in primary lung tumors. Elevated *BLIMP1* mRNA expression was detected in lung adenocarcinoma samples compared to normal lung tissues [49] (Fig. 2C). Constitutive Ras signaling induced by either a mutant *RAS* gene or upstream activator such as growth factor receptor has been implicated in many other tumors. *KRAS* mutations have been found in >95% of pancreatic ductal adenocarcinomas [50], while overexpression of Epidermal Growth Factor receptor (EGFR), which induces Ras signaling, was found in 80–90% human head and neck squamous cell carcinomas [51] and 40% of glioblastomas [52]. Notably, our analyses using microarray datasets in Oncomine revealed elevated *BLIMP1* RNA expression in samples of pancreatic adenocarcinoma [53], tongue squamous cell carcinoma [54] and glioblastoma [55] compared to the corresponding normal tissues (Fig. 2D). Thus, *BLIMP1* RNA is overexpressed in a diverse group of human cancers.

### Ras to c-Raf signaling induces Blimp1 expression in lung cancer cells

**Figure 3:**
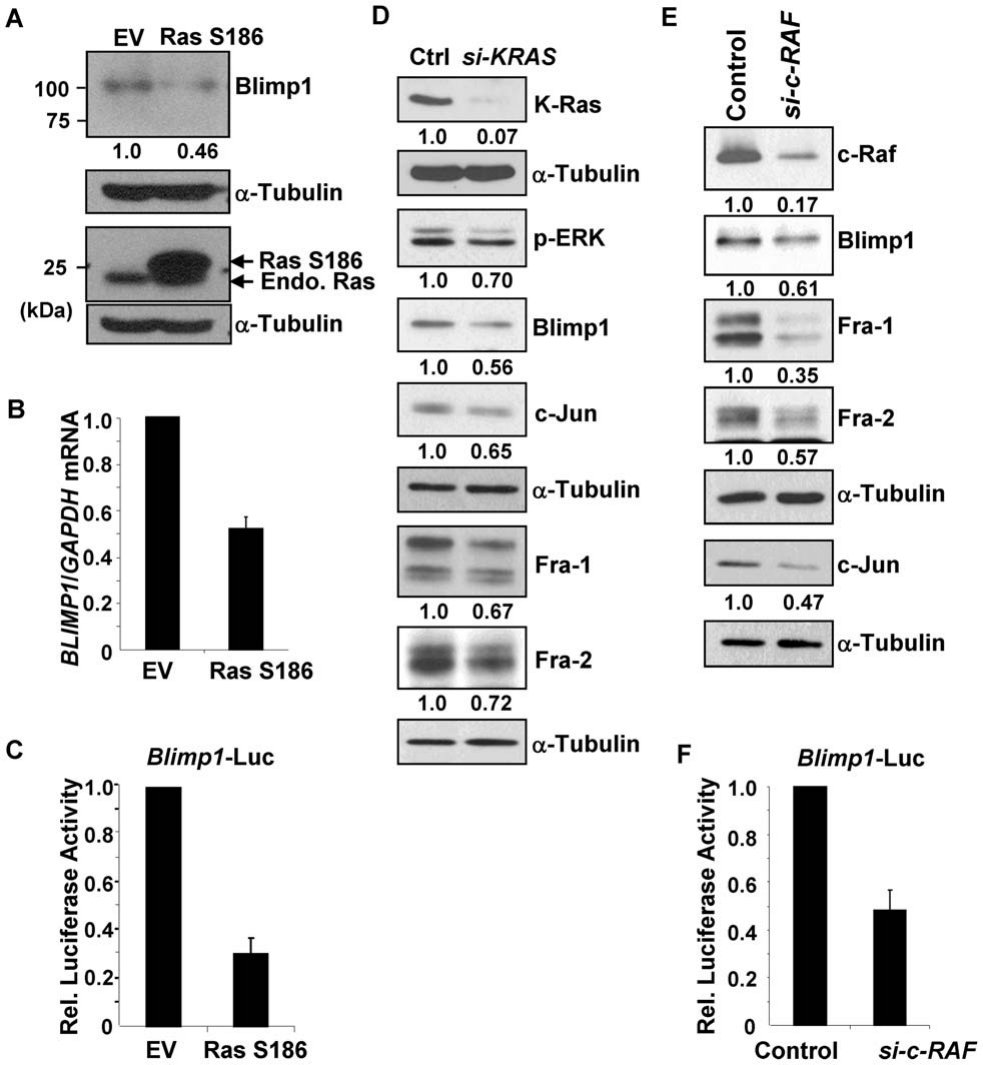
A Ras to c-Raf pathway induces the *Blimp1* promoter and AP-1 activity. (A) A549 cells were transfected with 5 µg of a plasmid expressing dominant negative Ras S186 or EV DNA. After 48 h, WCE and RNA were prepared. Samples (30 µg) of WCE were subjected to immunoblot analysis for Blimp1, Ras and α-tubulin. The bands were quantified using NIH Image J software and Blimp1 expression normalized to β-actin expression. The average values for normalized Blimp1 levels from two independent experiments are given relative to EV DNA (set to 1.0). (B) RNA was isolated from the A549 cells treated as in part A, and subjected to Q-PCR for *BLIMP1* mRNA and normalized to *GAPDH*. The values represent an average of two independent experiments. (C) A549 cells were transfected, in triplicate, with 0.16 µg of Ras S186 plasmid or EV DNA, 0.33 µg of a MSV- β-gal expression vector and 0.16 µg of the 7-kB *Blimp1* promoter *Blimp1*-Luc, in a 12-well plate. After 48 h, cell lysates were subjected to measurements for luciferase and β-gal activities and normalized *Blimp1* promoter activity values are presented as the mean ± SEM from two experiments (EV DNA set to 1.0). (D) Two-hundred pmol of an siRNA against K-Ras or a negative control siRNA (Ctrl) was incubated in the presence of 25 µl of Lipofectamine RNAiMAX in 2 ml of optiMEM in P100 plates. A549 cells (6.4×10^5^) were seeded at a final siRNA concentration of 20 nM for 48 h. WCE were subjected to immunoblotting for K-Ras, Blimp1, c-Jun, phospho-ERK (p-ERK), Fra-1, Fra-2, and α-tubulin. Average normalized levels of Blimp1, c-Jun, Fra-1, Fra-2 and K-Ras from two independent experiments are given relative to the control (set to 1.0). Immunoblots from one of two independent experiments with similar results are presented. (E) Two-hundred pmol of an siRNA against c-*RAF* or a negative control siRNA was incubated in the presence of 25 µl of Lipofectamine RNAiMAX in 2 ml of optiMEM in P100 plates. A549 cells (6.4×10^5^) were seeded at a final siRNA concentration of 20 nM for 48 h. WCE were subjected to immunoblotting for c-Raf, Blimp1, Fra-1, Fra-2, c-Jun, and α-tubulin. Average normalized levels of c-Raf, Blimp1, Fra-1, Fra-2 and c-Jun from two independent experiments are given relative to the control (set to 1.0). Immunoblots from one of two independent experiments with similar results are presented. (F) A549 cells were transiently transfected, in triplicate, with *si-c-RAF* or negative control siRNA at a final concentration of 20 nM in a 12-well plate. Eight h later, *Blimp1*-luc promoter construct (0.16 µg) and an MSV- β-gal expression vector (0.33 µg) were transfected into these siRNA-treated A549 cells for an additional 40 h. Relative (Rel.) *Blimp1* promoter activity values are presented as the mean ± SEM from two experiments (EV DNA set to 1.0).

To directly address the role of Ras signaling on Blimp1 levels in lung cancer cells, a dominant negative mutant was first used. The Ras S186 mutant retains the ability to associate with the effector protein kinase c-Raf but does not translocate to the membrane and inhibited activation of Blimp1 by Bcl-2 [13], [56]. A549 cells, which express an activated mutant K-Ras C12, were transfected with EV or a plasmid expressing Ras S186 and after 48 h, WCE and RNA were isolated. Ectopic expression of Ras S186, which was confirmed by immunoblotting, decreased Blimp1 protein expression by ∼54% (Fig. 3A). In two separate experiments, *BLIMP1* mRNA expression declined an average of 48% upon ectopic expression of Ras S186 (Fig. 3B). The effects of the dominant negative Ras on *Blimp1* promoter activity were also tested. A549 cells were co-transfected with either EV or Ras S186 vector DNA, along with a 7-kB *Blimp1* promoter reporter construct *Blimp1*-luc and a β-gal expression vector, for normalization of transfection efficiencies. Overexpression of Ras S186 led to an average decrease of 69% in normalized *Blimp1* promoter activity (Fig. 3C). Lastly, an si-*KRAS* strategy was employed (Fig. 3D). Knockdown of K-Ras led to a decrease of 93% of K-Ras protein expression and to a substantial decrease in ERK activity as judged by a reduction in phospho-ERK levels. Furthermore, an average decrease of 44% in Blimp1 levels were seen in two independent experiments (Fig. 3D). Together, these results indicate that oncogenic Ras signaling in A549 lung cancer cells drives *BLIMP1* gene expression.

Ras mediates its effects by signaling via several pathways. The c-Raf/Erk pathway has been implicated in control of migration and thus we used a knockdown strategy to test whether c-Raf mediates signals leading to induction of Blimp1. A549 cells were transfected for 48 h with a negative control siRNA or an siRNA against c-*RAF*, which effectively decreased levels of c-Raf and Blimp1 protein, which decreased by an average of 39% in two experiments (Fig. 3E). To confirm a role of c-Raf in *Blimp1* promoter activity, A549 cells were reverse-transfected with c-*RAF* siRNA or control siRNA, and after 8 h were transfected with a *Blimp1* reporter construct for 40 h. The *si-c-RAF* led to an average decrease of 52% in *Blimp1* promoter activity compared to negative control siRNA (Fig. 3F). Thus, a Ras to c-Raf pathway activates *BLIMP1* gene expression in A549 lung cancer cells.

### AP-1 induces Blimp1 expression

**Figure 4:**
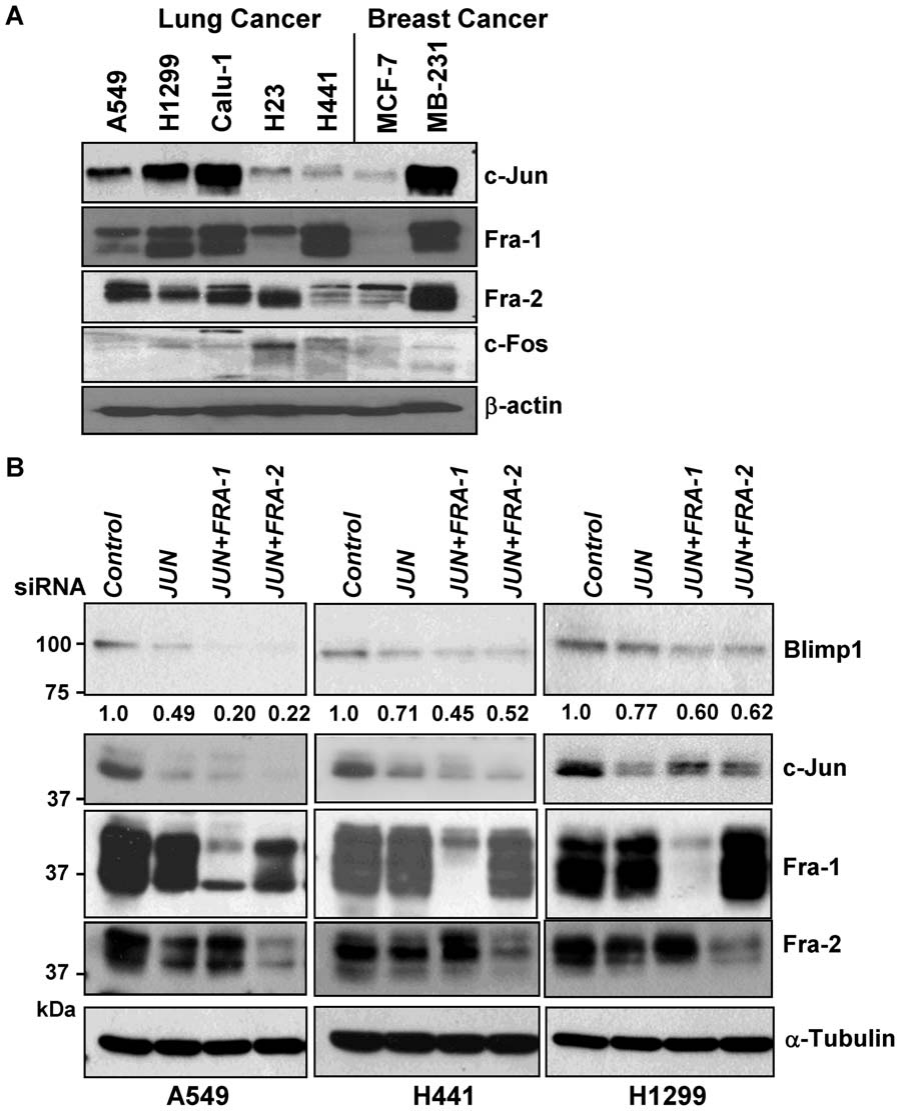
Knockdown of AP-1 subunits decreases Blimp1 expression in lung cancer cells. (A) The immunoblot of nuclear extracts from lung cancer cells in Fig. 1A was stripped and re-probed to assess expression of the AP-1 subunits c-Jun, Fra-1, Fra-2 and c-Fos. (B) A549, H441 and H1299 cells were transfected with 20 nM of *JUN* siRNA alone or 10 nM of *JUN* siRNA in combination with 10 nM of *FRA-1* or *FRA-2* siRNA or with 20 nM of a negative control siRNA (Qiagen) for 24 h. WCE (30 µg) were subjected to immunoblotting for Blimp1, c-Jun, Fra-1, Fra-2 and α-tubulin, as a loading control. The Blimp1 bands were quantified and normalized to α-tubulin expression, and average values from two independent experiments presented relative to control siRNA, set to 1.0.

The AP-1 family of transcription factors has been implicated in the highly migratory phenotype of lung cancer cells [19], [20], [21], and two functional AP-1 binding sites or TREs have been identified in the *BLIMP1* promoter [47]. Substantial decreases in amounts of c-Jun (53%), Fra-1 (65%) and Fra-2 (43%) resulted from treatment with the c-*RAF* siRNA (Fig. 3E), consistent with the observed reduction in Blimp1 expression. Similarly knockdown of Ras led to average decreases of 35, 33 and 28% in levels of c-Jun, Fra-1 and Fra-2, respectively (Fig. 3D). We next characterized AP-1 subunit expression in the lung cancer cell lines by subjecting nuclear extracts to immunoblot analysis (Fig. 4A). High levels of c-Jun were detected in H1299 and Calu-1 cells and low to moderate c-Jun levels in H23, H441 and A549 cells. High levels of Fra-1 were seen in H1299, Calu-1 and H441 cells, while A549 and H23 cells expressed low levels of Fra-1. All of the lung cancer cell lines except H441 expressed moderate to high levels of Fra-2, while only low levels of c-Fos were seen in all of the lines.

To test whether the c-Jun, Fra-2 and Fra-1 AP-1 subunits play a role in Blimp1 expression, a knockdown strategy was employed. The c-Jun subunit can form homodimers with Jun family members or heterodimers with Fos family members, while Fos family members only bind as heterodimers with Jun family members [57]. A549, H441 and H1299 cells were transfected with *JUN* siRNA alone or in combination with either *FRA-1* or *FRA-2* siRNA or a negative control siRNA. A *FOS* siRNA was not included as c-Fos expression appeared low in these lines. Effective knockdown of the corresponding AP-1 subunits in A549, H441 and H1299 cells was confirmed by immunoblot analysis (Fig. 4B). Depletion of c-Jun alone led to an average 51%, 29% and 23% decrease in Blimp1 expression in A549, H441 and H1299 cells, respectively. Simultaneous knockdown of c-Jun and Fra-1 led to more substantial decreases in Blimp1 expression of 80%, 55% and 40% in A549, H441 and H1299 cells, respectively. Knockdown of c-Jun and Fra-2 led to decreases in Blimp1 expression by 78%, 48% and 38% in A549, H441 and H1299 cells, respectively. These results indicate that AP-1 subunits c-Jun, Fra-1 and Fra-2 are all involved in the maintenance of basal Blimp1 expression in lung cancer cells.

**Figure 5:**
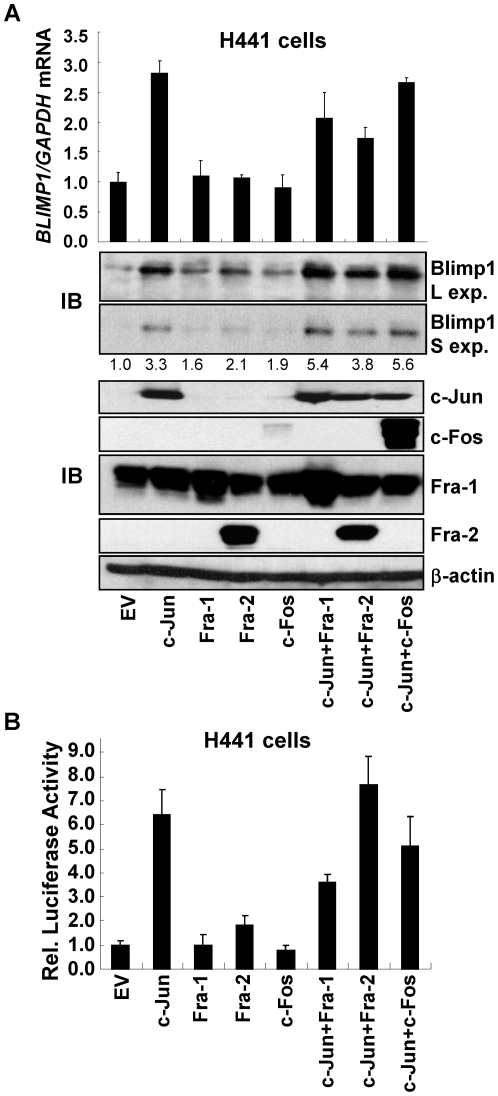
Ectopic AP-1 subunits induce Blimp1 expression. (A) H441 cells, growing in 6-well plates, were transfected with 1 µg of vectors expressing the indicated AP-1 subunits or EV DNA (see bottom) to make a 2 µg total. Upper panel. After 48 h, RNA was isolated and subjected to Q-PCR. The levels of *BLIMP1* mRNA normalized to *GAPDH* mRNA are presented as mean ± SD of three independent experiments. Middle and lower panels. WCE were isolated and subjected to immunoblotting (IB) for Blimp1 (Middle panels), and for c-Jun, Fra-1, Fra-2, c-Fos and β-actin (Lower panels). (L exp., longer exposure; S exp., shorter exposure). Blimp1 levels, normalized to β-actin, were determined as in Fig. 1C and average values from two independent experiments presented relative to EV DNA, set to 1.0. (B) H441 cells were transiently transfected, in triplicate, with 0.3 µg of *Blimp1*-Luc, 0.3 µg of MSV-β-gal, and vectors expressing the indicated AP-1 subunits (0.15 µg each) and EV DNA to a total of 1.0 µg DNA. Normalized values of *Blimp1* promoter activity are presented as the mean ± SEM from two experiments (EV DNA set to 1.0).

We next tested whether AP-1 complexes containing c-Jun with either Fra-1, Fra-2 or c-Fos induce Blimp1 expression and selected H441 cells, which express a low endogenous level of c-Jun. H441 cells were transfected with c-Jun, Fra-1, Fra-2 or c-Fos cDNA individually or in combination or with EV DNA for 48 h and subunit expression confirmed by immunoblotting (Fig. 5A, lower panels). Q-PCR was performed to measure the effects on *BLIMP1* mRNA expression. Data from three independent experiments show that relative to EV DNA, which was set to 1.0, ectopic expression of c-Jun, c-Jun-Fra-1, c-Jun-Fra-2 or c-Jun-c-Fos induced *BLIMP1* mRNA levels in H441 cells by an average of 2.8-, 2.1-, 1.7 or 2.6-fold, respectively (Fig. 5A, upper panels). Fra-1, Fra-2 and c-Fos alone had little effect on *BLIMP1* mRNA expression. Immunoblot analyses were quantified to assess the effects of these AP-1 subunits on Blimp1 protein and the average values from two independent transfection experiments were calculated relative to the EV DNA (Fig. 5A, middle panels). Ectopic expression of c-Jun alone induced endogenous Blimp1 protein expression 3.3-fold and the combinations of c-Jun with Fra-1, Fra-2 or c-Fos induced Blimp1 by an average of 5.4-, 3.8- or 5.6-fold, respectively. Fra-1, Fra-2 and c-Fos alone were again less effective (Fig. 5A, darker exposure, middle panels). Thus, AP-1 complexes containing c-Jun with Fra-1, Fra-2 or c-Fos, and possibly c-Jun homodimers, induce Blimp1 protein and mRNA expression in lung cancer cells.

Lastly, AP-1 factors were tested for their ability to induce *Blimp1* promoter activity in H441 lung cancer cells using a co-transfection assay. c-Jun alone or in combination with Fra-1, Fra-2 or c-Fos substantially induced normalized *Blimp1* promoter activity by an average of 6.4-, 3.6-, 7.7- or 5.1-fold, respectively (Fig. 5B). Expression of Fra-1, Fra-2, or c-Fos cDNA alone had only minor effects on the activity of the *Blimp1* promoter, as expected. Together, these results show that c-Jun containing AP-1 complexes (c-Jun-c-Jun, c-Jun-Fra-1, c-Jun-Fra-2 and c-Jun-c-Fos effectively induce *Blimp1* promoter activity, leading to elevated levels of Blimp1 expression.

### Ectopic c-Jun binds to the *BLIMP1* promoter

**Figure 6:**
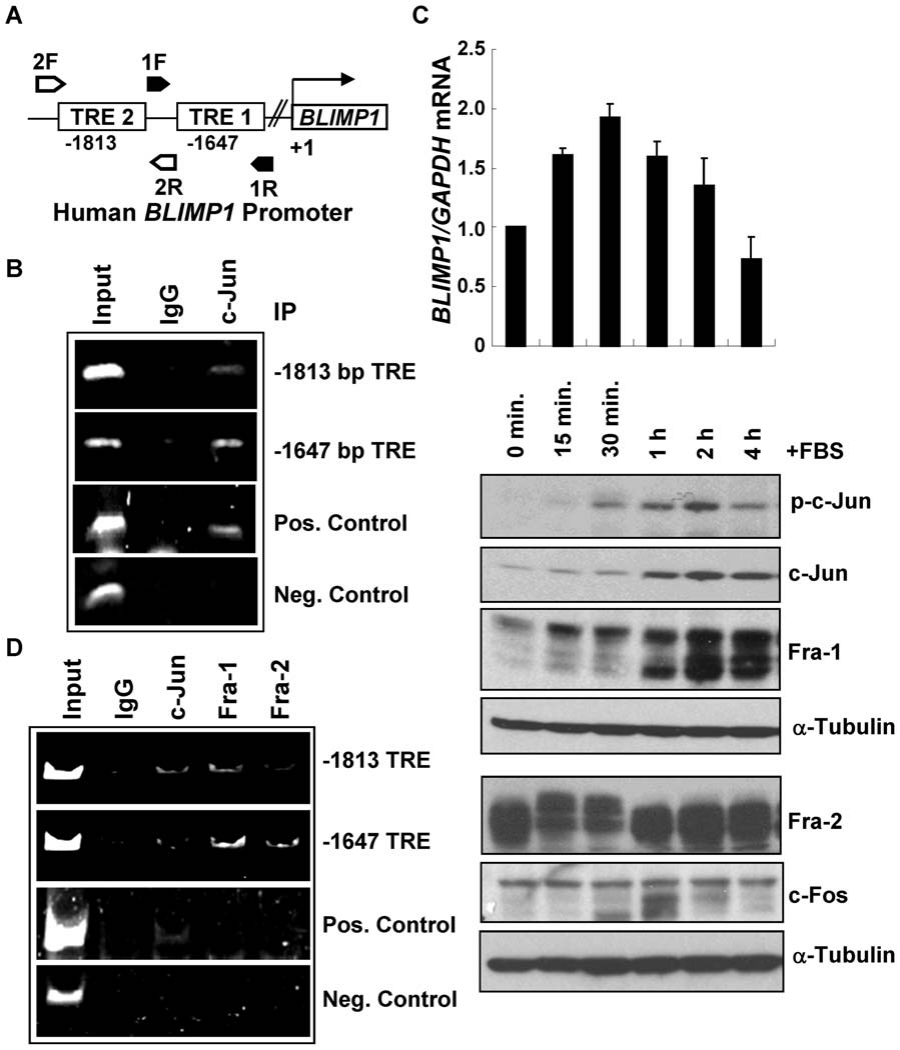
AP-1 subunits bind to the *BLIMP1* promoter. (A) Schematic of the localization of the two TRE sites on the human *BLIMP1* promoter. Two primer sets encompassing these sites are shown: 1F/1R amplifies the −1647 bp TRE and 2F/2R amplifies the −1813 bp TRE. (B) H441 cells at 90% confluence in P100 plates were transfected with 4 µg of c-Jun expression vector, and after 48 h subjected to a ChIP assay using a control IgG or c-Jun antibody, as described in the Materials and Methods. Input, 1% of the WCE. Positive (Pos.) control: a genomic region of the *JUN* promoter containing two TREs (−1 and −120 bp). Negative (Neg.) control: region upstream of *BLIMP1* transcription start site (∼−5.4 kB) that does not contain any known TRE sites. (C) A549 lung cancer cells were incubated in serum free DMEM for 48 h. FBS was added back to 10%. Samples were harvested at 0, 15, 30 minutes or 1, 2, 4 h and RNA and WCE prepared. Upper panel: RNA was subjected to Q-PCR, in triplicate, and values for *BLIMP1* normalized to *GAPDH* RNA levels presented relative to the 0 time point which was set to 1.0. Data for the mean ± SD from three independent experiments are presented. Lower panels: WCE (25 µg) were subjected to immunoblot analysis for phospho-c-Jun (p-c-Jun), c-Jun, Fra-1, Fra-2, c-Fos AP-1 subunits, and α-tubulin, which confirmed essentially equal loading control. Data shown is a representative of two independent experiments with similar results. (D) A549 cells were incubated in serum free DMEM for 48 h, and stimulated with addition of FBS (final 10%) for 30 min. Whole cell lysates were subjected to ChIP analysis using antibodies against c-Jun, Fra-1, Fra-2 or normal rabbit IgG, as described in part B. Data shown is a representative of two independent experiments with similar results.

Next, binding of AP-1 subunits to the *BLIMP1* promoter was examined using ChIP assays. The two identified AP-1 binding sites are located at −1647 and −1813 bp relative to the *BLIMP1* transcription start [47] (Fig. 6A). Since c-Jun plays an essential role in formation of homo- and hetero-dimers of AP-1 complexes, we first tested for direct binding of c-Jun to the TRE sites. H441 cells were transiently transfected with a c-Jun cDNA expression vector. ChIP analysis was performed using an anti-c-Jun or control IgG antibody, and resulting genomic DNA fragments analyzed by PCR (Fig. 6B). Amplification of the −1647 and −1813 bp TRE sites was observed using the two primer sets indicated in Fig. 6A. As a positive control, a primer set for previously described TREs on the *JUN* promoter was used [48]. Ectopically expressed c-Jun was also present on its own promoter (Fig. 6B), as expected. As a negative control, an upstream region of the *BLIMP1* promoter (−5508 to −5366 bp) without any known TRE consensus sequence was tested, and no amplification was observed following c-Jun antibody pull-down (Fig. 6B). These results indicate that the ectopically expressed AP-1 c-Jun subunit is recruited to the *BLIMP1* promoter in H441 lung cancer cells.

### Endogenous AP-1 subunits bind to the *BLIMP1* promoter

Our attempts to test for the binding of endogenous c-Jun to the *BLIMP1* promoter in A549 lung cancer cells cultured in growth medium supplemented with 10% FBS were unsuccessful as no amplification of either the *BLIMP1* or positive control *JUN* TRE sites was detected in ChIP analyses (data not shown). Since AP-1 activities are controlled by Ras-MAPK signaling and because AP-1 subunits have been shown to be recruited to target gene promoters upon serum stimulation [58], [59], we tested the temporal induction of *BLIMP1* mRNA levels, and AP-1 expression as a function of time after serum stimulation. A549 lung cancer cells were incubated for 48 h in serum free DMEM medium. FBS was added back and proteins and mRNA isolated after 0, 15, or 30 min or 1, 2, or 4 h. The expression of AP-1 subunits was monitored. Phospho-c-Jun levels began to increase by the 15 min time point and peaked at 2 h, while a substantial increase in total c-Jun levels was noted at 1 h (Fig. 6C, lower panels). A markedly slower migration of Fra-2 bands was noted by 15 min, presumably active phosphorylated forms, which lasted until 30 min. An increase in the slower migrating, presumably phosphorylated, Fra-1 was also seen at 15 min, and these levels remained high throughout the time course. An increase in total Fra-1 levels was initially observed at the 1 h time point. Levels of c-Fos remained relatively low but increased at 1 h. Thus, serum rapidly induces AP-1 phosphorylation and a later increase in total AP-1 expression levels. RNA, which isolated over the same time course, was subjected to Q-PCR assays for *BLIMP1* and *GAPDH*, as loading control. The normalized levels of *BLIMP1* mRNA increased within 15 min after serum stimulation, peaked at ∼2-fold at 30 min and stayed elevated until 2 h (Fig. 6C, upper panel). By the 4 h time point, *BLIMP1* mRNA levels were low, suggesting a rapid but transient transcriptional activation. The 30 min time point was selected to assess for induction of AP-1 binding to the *BLIMP1* promoter.

To test whether the rapid induction of AP-1 activity is responsible for the increase of *BLIMP1* mRNA, A549 cells were serum deprived, and then stimulated with FBS for 30 min. ChIP analysis was performed using antibodies against c-Jun, Fra-1, or Fra-2 or the pre-immune IgG. PCR amplification of the −1647 and −1813 bp TRE sites of the *BLIMP1* promoter was observed with c-Jun, Fra-1 or Fra-2 antibodies (Fig. 6D). The positive control TRE sites on the *JUN* promoter were also amplified with the antibody against c-Jun, consistent with the literature [48]. We were unable to detect precipitation of the *JUN* DNA with Fra-1 or Fra-2 antibodies. No amplification of the negative control region of the *BLIMP1* promoter was observed. Taken together, these experiments show that endogenous c-Jun, Fra-1 and Fra-2 AP-1 subunits are recruited to the *BLIMP1* promoter upon serum stimulation, further implicating binding of AP-1 subunits to the TRE sites in regulation of Blimp1 expression.

### LOX-PP reduces the expression of Blimp1 and its upstream AP-1 activators

**Figure 7:**
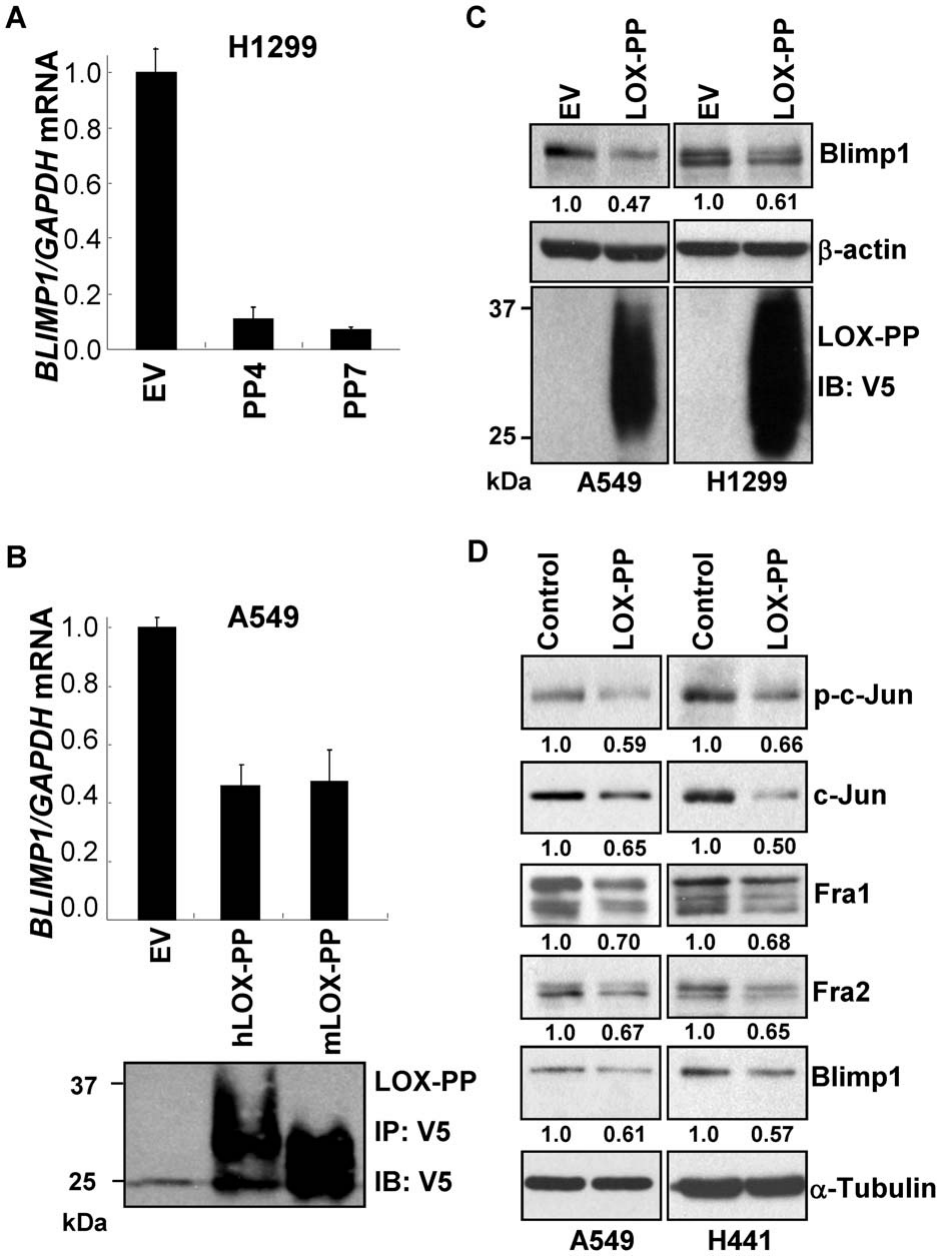
Ectopic LOX-PP reduces Blimp1 expression in lung cancer cells. (A) H1299-EV cells, and H1299-LOX-PP4 (PP4) and H1299-LOX-PP7 (PP7) clones, isolated as described previously [25], were treated in triplicate with 2 µg/ml dox for 48 h. RNA from two independent experiments was subjected to Q-PCR and normalized values for *BLIMP1* mRNA relative to *GAPDH* levels are presented as the mean ± SEM (EV DNA set to 1.0). (B) A549-EV, A549-hLOX-PP, A549-mLOX-PP dox-inducible stable populations were treated with 2 µg/ml dox for 48 h in DMEM supplemented with 0.5% FBS. FBS was added back to 10% and cells incubated overnight. RNA from two independent experiments was subjected to Q-PCR and normalized values for *BLIMP1* mRNA relative to *GAPDH* levels are presented as the mean ± SEM (EV DNA set to 1.0). Samples of medium (5 ml) were subjected to immunoprecipitation followed by immunoblotting using V5 antibody for LOX-PP expression. (C) A549 and H1299 cells were transiently transfected with human LOX-PP cDNA or EV DNA. After 48 h, media and WCE were prepared. Samples of media (50 µl) were subjected to immunoblotting for V5. Samples of WCE (25 µg) were probed for Blimp1 and β-actin, and average normalized Blimp1 values from two independent experiments presented relative to EV DNA, set to 1.0. (D) A549 and H441 cells were treated with purified recombinant LOX-PP protein at a final concentration of 4 or 1 µg/ml, respectively, or the same volume of vehicle (water) in medium with 0.5% FBS. Twenty-four h later, FBS was added back to 10% and cultures incubated overnight. WCE were subjected to immunoblotting for Blimp1, phospho-c-Jun (p-c-Jun), total c-Jun, Fra-1 and Fra-2 and α-tubulin, as a loading control. Normalized Blimp1 and AP-1 subunit values from two independent experiments are presented relative to EV DNA, set to 1.0.

As the amino terminal LOX-PP domain of Pro-LOX has the ability to inhibit Ras-mediated transformation of NIH 3T3 cells [32] and H1299 lung cancer cells [25], its ability to reduce Blimp1 was next examined. We first tested the effects of induction of LOX-PP in two stable H1299 tet-on clones expressing ectopic LOX-PP, which were described previously [25]. A robust decrease in *BLIMP1* RNA (∼90%) was observed upon induction of LOX-PP, as judged by Q-PCR (Fig. 7A). To extend the findings to a second line, stable A549 cell populations expressing either human LOX-PP (hLOX-PP) or mouse LOX-PP (mLOX-PP) in a dox-inducible vector or with EV DNA were prepared. Following incubation in the presence of dox for 48 h, induction of ectopic human or mouse LOX-PP expression was found to reduce *BLIMP1* mRNA by an average of ∼60% compared to EV DNA (Fig. 7B). Ectopic LOX-PP expression was confirmed by immunoblotting (Fig. 7B). The effects of LOX-PP on Blimp1 protein expression were further analyzed by transiently transfecting A549 and H1299 cells with LOX-PP cDNA. Ectopic expression of LOX-PP was confirmed by immunoblotting (Fig. 7C). An average decrease of 53 and 39% in Blimp1 expression was observed in A549 and H1299 cells, respectively.

We next assessed the ability of LOX-PP to reduce the induction of AP-1 subunits and Blimp1 mediated by serum. H441 and A549 cells were serum starved for 24 h in culture medium with 0.5% FBS in the presence of 1 or 4 µg/ml purified functionally active recombinant LOX-PP protein (rLOX-PP), respectively, prepared as reported previously [34], [60]. FBS was added back to a final concentration of 10% and cultures incubated for another 16 h (Fig. 7D). Notably, the levels of active phospho-c-Jun were decreased by an average of 41% and 34% by LOX-PP treatment in A549 and H441 cells, respectively. Total c-Jun was decreased by an average of 35% and 50%, respectively in the LOX-PP-treated A549 and H441 cells. Fra-1 expression was decreased by an average of 30–32% and an average decrease of 33–35% in Fra-2 expression was observed in these two cell lines. A commensurate decrease in Blimp1 levels resulted from LOX-PP treatment (∼40% in A549 and H441 cells) (Fig. 7D). Thus LOX-PP reduces the increase in Blimp1 expression and its upstream activators c-Jun, Fra-1 and Fra-2 following serum stimulation.

### LOX-PP-mediated reduction in lung cancer cell migration occurs via repression of Blimp1

**Figure 8:**
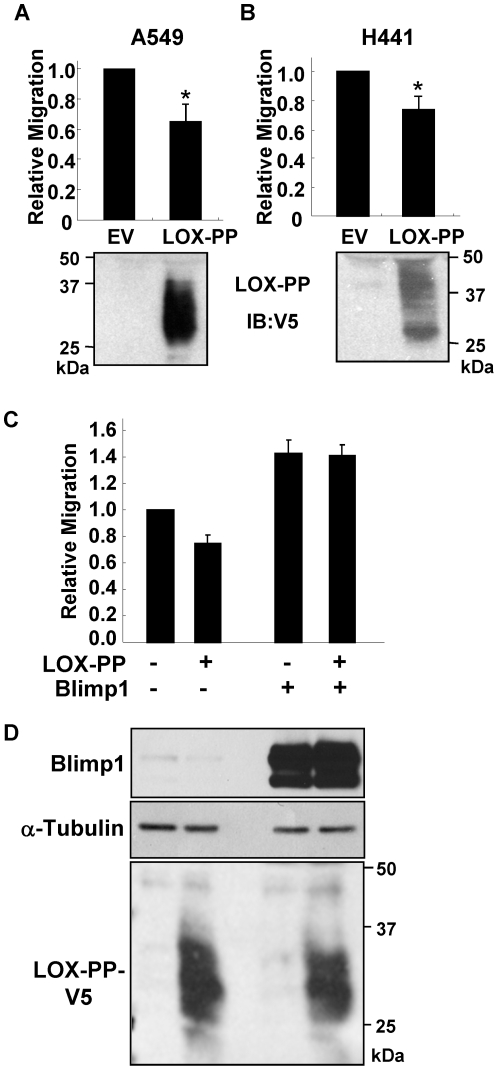
LOX-PP represses the migratory phenotype of lung cancer cells via inhibiting Blimp1. (A) A549 and (B) H441 cells were transfected with 2 µg of EV or human LOX-PP cDNA. Upper panels: After 24 h, 1×10^5^ transfected cells were subjected to migration assay. Lower panels: After 48 h, culture media was isolated and samples (50 µl of 2 ml total) subjected to immunoblotting using an anti-V5 antibody for LOX-PP. (C and D) H441 were transiently transfected with 1 µg of LOX-PP or Blimp1 DNA alone or in combination, or EV DNA (2 µg total DNA). (C) After 24 h. cells were subjected to migration assays, in triplicate, for 16 h. The average migration from two independent experiments ± SEM is presented relative to the EV (set at 1.0). (D) WCE and media were isolated. WCE samples (25 µg) were subjected to immunoblotting for Blimp1 and α-tubulin. Media samples (50 µl) were subjected to immunoblotting for LOX-PP-V5. Immunoblots from one of two independent experiments with similar results are presented.

To test the effects of LOX-PP on lung cancer cell migration, A549 and H441 cells were transiently transfected with a LOX-PP expressing cDNA or EV DNA. After 24 h, cells were subjected to a migration assay. LOX-PP expression led to an average decrease of 39% and 26% in migration of A549 and H441 cells, respectively, compared to EV (Fig. 8A and 8B, upper panels). Expression of LOX-PP in the media was confirmed by immunoblot analysis (Fig. 8A and 8B, lower panels). Similar results were also observed with addition of rLOX-PP, which resulted in an ∼40% decrease in migration (data not shown).

Next, in order to assess the role of Blimp1 in LOX-PP-mediated decrease in lung cancer cell migration, we asked whether ectopic Blimp1 expression can override the observed inhibition. H441 cells were transiently transfected with EV DNA or LOX-PP in the absence or presence of Blimp1 cDNA and subjected to migration assays. Ectopic LOX-PP decreased endogenous Blimp1 expression and led to a 26% reduction in cell migration while ectopic Blimp1 increased cell migration by 43% (Fig. 8C), consistent with the earlier findings. Importantly, LOX-PP-transfected cells expressing ectopic Blimp1 displayed an ability to migrate at a level similar to those expressing ectopic Blimp1 only. Immunoblot analysis confirmed ectopic expression of LOX-PP and Blimp1 expression (Fig. 8D). Together these results argue for a role of repression of Blimp1 expression in the inhibition of migration by LOX-PP.

### Physical interaction of LOX-PP with c-Raf inhibits Blimp1-mediated cell migration

**Figure 9:**
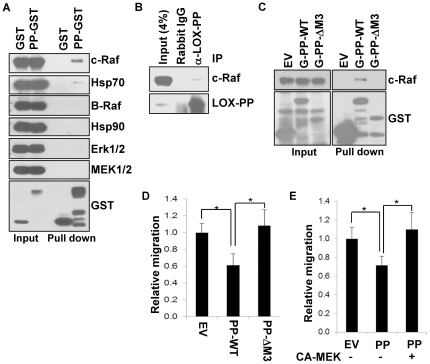
LOX-PP inhibits the migratory phenotype of lung cancer cells via interaction with c-Raf. (A) H1299 cells were transfected with expression plasmids for GST or LOX-PP-GST (PP-GST) and tagged proteins purified on Glutathione-Sepharose 4B beads. Bound proteins were analyzed by immunoblotting for the indicated proteins. For estimation of the amounts of expressed proteins, 4% of each of the lysates was immunoblotted (Input). (B) Triton X-100 extracts of A549 were immunoprecipitated with the indicated antibodies. The precipitated proteins were analyzed by Western blotting for c-Raf and LOX-PP. As the band of precipitated LOX-PP migrated close to that of rabbit IgG light chain, Protein A-conjugated HRP was used as a secondary ‘antibody’ to detect immunoprecipitated LOX-PP. (C) GST (EV), GST-LOX-PP WT (G-PP-WT), or GST-LOX-PP ΔM3 (G-PP-ΔM3) were expressed in H1299 cells for 24 h and tagged proteins purified as in part A and subjected to Western blotting with the indicated antibodies. Input = 4% of the lysate. (D) Either GST (EV), GST-LOX-PP WT or GST-LOX-PP ΔM3 (Δ26–100) were expressed in H1299. After 24 h, cells were subjected to a migration assay in triplicate for 16 h. Cells that migrated to the lower side of the filter were stained and quantified by spectrometric determination at *A_570 nm_*. The average values from three independent experiments ± SD presented relative to the EV control, set to 1.0. *P* values were calculated from three independent experiments using Student's t test. *, P<0.01. (E) GST (EV) or GST-LOX-PP (PP) was co-transfected in H1299 cells in presence of a vector expressing a constitutively active MEK mutant (CA-MEK) or EV DNA (−) for 24 h. Cultures were subjected to a migration assay for 16 h, in triplicate, as in part D. *P* values of three independent experiments were calculated using Student's t test. *, P<0.01.

We recently noted that LOX-PP can physically interact with c-Raf and Hsp70 in breast cancer cells [61]. Given the role of c-Raf in activation of Blimp1, their physical association in lung cancer cells was next tested. Association of c-Raf with LOX-PP in the H1299 lung cancer line was monitored using GST-pull down assays. Cells were transfected with a vector expressing LOX-PP-GST or GST protein. LOX-PP-GST brought down c-Raf and Hsp70, but not B-Raf, Hsp90, Erk1/2 and MEK1/2 (Fig. 9A). Triton X-100 soluble lysates of A549 cells were immunoprecipitated with an antibody against either LOX-PP or rabbit control IgG. The antibody against LOX-PP brought down the LOX-PP peptide as well as c-Raf, confirming the ability of these endogenous proteins to interact (Fig. 9B). The region of LOX-PP encompassing aa 26 to 100 is necessary for its interaction with c-Raf in breast cancer cells [61]. Lysates were prepared from H1299 cells ectopically expressing either GST, GST-LOX-PP WT or GST-LOX-PP ΔM3 (with a deletion of aa 26–100) and subjected to purification with Glutathione-Sepharose 4B beads. Binding of c-Raf to full-length LOX-PP was readily detected, whereas no binding was seen with the GST-LOX-PP ΔM3 protein, or with the GST control protein (Fig. 9C). These results suggest that aa 26 to 100 of LOX-PP is necessary for its interaction with c-Raf in H1299 cells. To assess the role of LOX-PP and c-Raf interaction in migration, assays were performed 24 h after ectopic expression of GST, full-length GST-LOX-PP WT, or GST-LOX-PP ΔM3 in H1299 cells. An approximately 40% reduction was seen with expression of full-length LOX-PP. In contrast, no reduction in H1299 cell migration was seen with the LOX-PP ΔM3 mutant (Fig. 9D). Lastly, a constitutively active mutant of MEK, which is downstream of c-Raf, was able to override the inhibition of migration by LOX-PP (Fig. 9E), confirming the importance of this pathway. Thus, the domain comprising aa 26 to 100, which mediates the interaction of LOX-PP with c-Raf, is required for inhibition of migratory activity.

## Discussion

Here we demonstrate that Blimp1, the zinc finger master regulator of B and T cells, is aberrantly activated in lung cancer cells by the oncogenic Ras/c-Raf to AP-1 pathway, and functions to promote their migratory phenotype. Furthermore, high *BLIMP1* RNA typifies several other aggressive cancers frequently driven by Ras signaling, including pancreatic and head and neck carcinomas as well as glioblastomas. The anti-cancer peptide LOX-PP, which was found to interact with c-Raf in lung cancer cells, repressed the induction of AP-1. Blimp1, which was detected in all five lung cancer cell lines examined, promoted lung cancer cell migration as judged by both knockdown and ectopic expression approaches. The ability of a dominant-negative Ras (Ras S186), and of knockdown of K-Ras and c-Raf and multiple AP-1 subunits to decrease Blimp1 levels in lung cancer cells confirmed the role of this signaling axis in aberrant expression of this zinc finger protein. Notably, c-Raf has recently been found essential for development of K-Ras-driven NSCLCs [62]. The ability of LOX-PP to inhibit this pathway and the observation that lung cancers are typified by greatly reduced levels of *LOX* gene expression [24], [25], suggests the potential use of LOX-PP in therapy of these cancers.

Mechanistically, Blimp1 expression is shown here to be positively regulated by AP-1 subunits. To our knowledge, this is the first study showing the induction of Blimp1 expression by AP-1 factors in epithelial cancer cells. Of note, deregulated AP-1 activity alone is sufficient for neoplastic transformation and critically necessary for the function of upstream dominant oncogenes, including members of growth factor receptor family which signal via the Ras-MAPK system [63]. AP-1 subunits including c-Jun, c-Fos and Fra-1 have been implicated in promoting cell motility in lung cancer [20], [64], [65]; although, the downstream targets were not fully elucidated. The c-Fos AP-1 subunit was shown to induce accelerated expression of Blimp1 upon CD40L/IL-4 treatment of B cells [66]. Our data implicate c-Jun containing complexes (c-Jun-c-Jun, c-Jun-Fra-1, c-Jun-Fra-2 and c-Jun-c-Fos) in Blimp1 expression in lung cancer cells. The observation that c-Jun, Fra-1 and Fra-2 AP-1 subunits are physically present on the TRE sites of the *BLIMP1* promoter indicates their direct control of transcription following growth factor stimulation. Activation of ERK has been found to lead to c-Jun phosphorylation at Ser 63 and 73 [67], [68] which subsequently upregulates c-Jun expression via a positive feed-back loop. In addition, Fra-1 and Fra-2 are directly activated by ERK which may enhance their DNA binding in conjunction with c-Jun [69]. These findings suggest that the Ras downstream effectors c-Jun, Fra-1 and Fra-2 are all involved in the expression of Blimp1 in lung cancer cells.

Microarray data from Oncomine confirmed upregulated *BLIMP1* mRNA expression is present in lung, pancreatic, head and neck cancers, and glioblastomas. In addition to oncogenic Ras mutations, 15–30% of samples of NSCLC, which make up 85% of total lung cancers, were also found positive for overexpression of EGFR [70], which signals via Ras, and has been implicated in their increased ability to invade and metastasize [70]. It is known that Ras mutations, especially activating K-Ras mutations, occur in more than 95% of pancreatic cancers [50]. High frequencies of EGFR overexpression have been reported in head and neck squamous cell carcinomas [51] and glioblastomas [52]. All these evidences suggest a role for Blimp1 as a mediator of the aberrantly activated Ras/MAPK signaling pathway under pathological conditions. Although Blimp1 may play an important etiologic role in development of these cancers in vivo, unfortunately, as was found with NF-κB, it cannot likely serve as a direct therapeutic target for most cancers given its essential role in directing the immune response. However, we hypothesized that as Blimp1 can only repress genes that are being actively expressed, that a distinct subset of targets will exist in transformed epithelial vs immune cells. Our microarray data in breast cancer cells has confirmed this hypothesis (Mathilde Romagnoli and G.E.S., unpublished observations). Together, these findings suggest that the observed aberrant expression of Blimp1 in lung and other epithelial cancers may have important clinical ramifications, leading to development of new therapeutic modalities.
